# Evaluating immunohaematological profile among COVID-19 active infection and recovered patients in Ghana

**DOI:** 10.1371/journal.pone.0273969

**Published:** 2022-09-12

**Authors:** Yatik Konlaan, Samuel Asamoah Sakyi, Kwame Kumi Asare, Prince Amoah Barnie, Stephen Opoku, Gideon Kwesi Nakotey, Samuel Victor Nuvor, Benjamin Amoani

**Affiliations:** 1 Department of Microbiology and Immunology, School of Medical Sciences, College of Health and Allied Sciences, University of Cape Coast, Cape Coast, Ghana; 2 Department of Molecular Medicine, School of Medicine and Dentistry, Kwame Nkrumah University of Science and Technology, Kumasi, Ghana; 3 Department of Biomedical Sciences, College of Health and Allied Sciences, School of Allied Health Sciences, University of Cape Coast, Cape Coast, Ghana; 4 Department of Forensic Science, School of Biological Sciences, College of Agriculture and Natural Sciences, University of Cape Coast, Cape Coast, Ghana; Tanta University Faculty of Medicine, EGYPT

## Abstract

**Introduction:**

The rapid spread of COVID-19 has been a global public health problem and it is yet to be put under control. Active COVID-19 is associated with unrestrained secretion of pro-inflammatory cytokines and imbalances in haematological profile including anaemia, leukocytosis and thrombocytopaenia. However, the haematological profile and immune status following recovery from COVID-19 has not been recognized. We evaluated the immunohaematological profile among COVID-19 patients with active infection, recovered cases and unexposed healthy individuals in the Ashanti region of Ghana.

**Methodology:**

A total of 95 adult participants, consisting of 35 positive, 30 recovered and 30 unexposed COVID-19 negative individuals confirmed by RT-PCR were recruited for the study. All the patients had the complete blood count performed using the haematological analyzer Sysmex XN-1500. Their plasma cytokine levels of interleukin (IL)-1β, IL-6, IL-10, IL-17, tumour necrosis factor-alpha (TNF-α) and interferon gamma (IFN-γ) were analysed using ELISA. Statistical analyses were performed on R statistical software.

**Result:**

The Patients with COVID-19 active infection had significantly higher levels of IL10 (181±6.14 pg/mL vs 155.00±14.32 pg/mL vs 158.80±11.70 pg/mL, *p* = 0.038), WBC count (5.5±0.4 x10^9^ /L vs 4.5±0.6 x10^9^ /L vs 3.8±0.5, *p* < 0.0001) and percentage basophil (1.8±0.1% vs 0.8±0.3% vs 0.7±0.2%, *p =* 0.0040) but significantly lower levels of IFN-γ (110.10±9.52 pg/mL vs 142.80±5.46 pg/mL vs 140.80±6.39 pg/mL, *p =* 0.021), haematocrit (24.1±3.7% vs 38.3± 3.0% vs 38.5±2.2%, *p* < 0.0001), haemoglobin concentration (9.4±0.1g/dl vs 12.5± 5.0g/dl vs 12.7±0.8, *p* < 0.0001) and MPV (9.8±0.2fL vs 11.1±0.5fL vs 11.6±0.3fL, *p* < 0.0001) compared to recovered and unexposed controls respectively. There were significant association between IL-1β & neutrophils (*r* = 0.42, *p*<0.05), IL-10 & WBC (*r* = 0.39, *p*<0.05), IL-10 & Basophils (*r* = -0.51, *p*<0.01), IL-17 & Neutrophil (*r* = 0.39, *p*<0.05) in the active COVID-19 cases.

**Conclusion:**

COVID-19 active infection is associated with increased IL-10 and WBC with a concomitant decrease in IFN-γ and haemoglobin concentration. However, recovery from the disease is associated with immune recovery with appareantly normal haematological profile.

## Introduction

The infection of Severe Acute Respiratory Syndrome Coronavirus 2 (SARS-CoV-2), the virus that causes COVID-19 is a pandemic that has battled the world for more than a year now with no indication of backing down [1, 2]. COVID-19 exhibits a range of symptoms from asymptomatic to acute respiratory distress syndrome (ARDS) [3]. Currently, there are effective vaccines to protect the people from COVID-19 and there have been a significant reduction in the global COVID-19 cases, emergency department visits, hospital admissions and mortality following the introduction of vaccines [4]. This has resulted in the reduction of COVID-19 restrictions such as mandatory wearing facemasks and social distancing.

The deadly form of COVID-19 have been associated with the development of cytokine storms, especially in comorbidities [5]. The unrestrained secretion of TNF-ɑ, IL-1, IL-6, and IFN-γ are some of the pro-inflammatory cytokines underlying the pathogenesis of the severe and life-threatening disease conditions in COVID-19 [6, 7]. The high elevated levels of IL-6, IL-1 and TNF-ɑ have been associated with the increased monocytes and macrophages in the COVID-19 patients experiencing cytokine storm [5, 8].

The immune system underlying COVID-19 and its complications are not fully elucidated. The peripheral blood circulation of natural killer (NK) cells, macrophages, monocytes, granulocytes, eosinophils, dendritic cells, Basophils and neutrophils which are major components of the innate immune system that expresses several varieties of pattern recognition receptors (PRRs) responsible for identifying pathogen-associated molecular patterns (PAMPs) during infections have been reported in COVID-19 cases [9, 10]. The recognition of PRRs such as toll-like receptors by PAMPs on the infecting pathogens triggers the expression of NF-kB, a pro-inflammatory cytokine transcription factor responsible for activating type 1 interferons against viral infection [11, 12]. The type 1 interferons stimulate the NK cells and CD8 cytotoxic T cells. NK cells recognize infected cells that downregulate MHC class 1 on their surface and upregulate stress ligands, and secrete perforin and granzyme to induce apoptosis. On the other hand, CD8+ T cells recognize viral peptides on MHC class 1 on infected cells and along with co-stimulation also secrete perforin-mediated granulysin to their cellular targets [13, 14].

However, in severe COVID-19 cases, the NK and the cytolytic CD8 T cells are not able to kill the SARS-CoV-2 resulting in the over activation of the antigen-presenting cells and exaggerated interactions leading to cytokine storms, thrombotic tendency, multiple organ failure and death [15, 16]. The monocytes and macrophages inflammatory cells infiltrate the lungs and cause alveolar injury [17, 18]. The hyperstimulation and infiltration of monocytes and macrophages into the organs in SARS-CoV-2 infections result in a condition called macrophage activation syndrome (MAS) [19]. The MAS is characterised by fever, pancytopenia, elevated serum ferritin, liver dysfunction, and splenomegaly which impairs viral clearance [15, 19]. Also, the increase in neutrophil infiltration in the organs in COVID-19 has been suggested to play a role in COVID-19 pathogenesis by forming neutrophil extracellular traps (NETs) and NETosis, through a programmed cell death in apoptosis and necrosis [15, 20–23].

Haematological parameters have been reported in active COVID-19 [24]. Anaemia, decrease in the erythropoiesis and mean platelet volume (MPV) have been associated with mild to severe COVID-19 cases. Multiple unknown factors may be contributing to anaemia in critically ill patients suffering from COVID-19 [25, 26]. Monitoring the haematological abnormalities in COVID-19 is critical for clinical prognosis and prompt management of affected patients [27, 28]. Moreover, thrombocytopenia is commonly reported in active COVID-19 and is significantly associated with death [29]. Again, high white blood cell (WBC) and thrombocytopenia has been described among patients with severe and fatal COVID-19 [30]. Monitoring these parameters following recovery from COVID-19 may be required to make decisions such as the need to continue treatment following discharge from the hospital [31].

The immunohaematological profile has been well studied in active COVID-19. However, there is paucity of data on the haematological profile and immune status following COVID-19 recovery. We evaluated the immunohaematological profile among COVID-19 patients with active infection, recovered cases and unexposed healthy individuals in the Ashanti region of Ghana.

## Materials and methods

### Participants, study design, and data collection, ethics & consents

This case-control study was conducted in the Kumasi Metropolis in the Ashanti Region of Ghana from January 2021 to June 2021. A total of 95 unvaccinated adult participants, consisting of 35 COVID-19 positive participants with active infection, 30 recovered patients and 30 unexposed COVID-19 negative controls were recruited for the study. Positive group consisted of patients who had been diagnosed and confirmed to be positive for COVID-19 by Reverse Transcription-quantitative Real Time-Polymerase Chain Reaction (RT-qPCR) [32]. The recovered group consisted of individuals who had been previously confirmed to be positive for SARS-CoV-2 nucleic acid by RT-qPCR, undergone treatment in hospital and had been discharged according to standard protocols. After two weeks of isolation, these patients were regarded as completely recovered, with no SARS-CoV-2 remaining in the body and a negative nucleic acid detection test. The unexposed negative controls consisted of individuals who had tested negative for SARS-CoV-2 by RT-qPCR. Non exposure among the controls was confirmed by periodical RT-qPCR testing for a period of three months. Participants in each of the study groups were excluded if they had known chronic or inflammatory condition such as HIV, tuberculusis, Asthma, diabetes and hypertension. This study was submitted to and approved by Committee on Human Research Publication Ethics (CHRPE), KNUST (CHRPE/AP/238/20). Written informed consent was sought from all participants who opted to particiapte after the aims and objectives of the study had been explained to them. All study participants were 18 years or older and did not require consent from parent/guardian to participate. Participation was voluntary and respondents were assured of data confidentiality. All methods were carried out in accordance with relevant guidelines and regulations.

### Laboratory examination of blood samples

Approximately 3 to 5 mL of peripheral blood was obtained with EDTA collection tubes from the subjects in each study group. Testing for hematological parameters (White blood cell, Basophil, Eosinophil, Monocyte, Lymphocyte, Neutrophil, Platelet, MCHC, MCH, MCV, MPV, Hematocrit, Hemoglobin and Red blood cell) was performed using a hematological analyzer Sysmex XN-1500 (Sysmex Corp., Kobe, Japan).

### Enzyme Linked Immunosorbent Assay (ELISA)

Enzyme Linked Immunosorbent Assay (ELISA) was used to detect the cytokine concentration [pg/mL] in the plasma samples including: IL-1β, IL-6, IL-10, IL-17, IFN-γ and TNF-α according to the manufacturer’s instruction. ELISA kit used was by shanghai Group Company limited.

### Statistical analyses

All statistical analyses were performed using the R language for statistical computing [33]. Continous data were expressed as mean±standard deviation whilst categorical variables were expressed as frequencis and percentages. The One-way Analysis of Variance (ANOVA) and Tukey multiple comparism test was used to compared haematologcal parameters and cytokine levels among the three study groups. Pearson’s correlation test was performed to test association between white cell parameters and cytokine levels. Statistical significance was determined as p < 0.05.

## Results

### Demographic characteristics of the study subjects

A total of 95 participants,18–69 years, consisting of 35 COVID-19 active cases, 30 COVID-19 recovered patents and 30 unexposed healthy controls were included in the statistical analysis. There was no significant difference in the mean age between the COVID-19 active cases, unexposed and the recovered (35.8±12.0 years vs 39.7±13.3 years vs 44.6±13.3 years, *p* = 0.145). The distribution of males and females were similar among the three study groups (*p* = 0.820) (Table 1).

**Table 1:** Demographic characteristics among the study participants.

		COVID-19 STATUS			
Variable	N	Active (35)	Unexposed (30)	Recovered (30)	*p*-value
Age (years)	95	35.8±12.0	39.7±13.3	44.6±13.3	0.145^b^
**Gender**					0.820^a^
Male	55	20 (57.4%)	17 (56.7%)	18 (60.0%)	
Female	40	15 (42.6%)	13 (43.3%)	12 (40.0%)	

N = number of participants. ^a^ *p*-values are chi-square test of association, ^b^ *p*-values were obtained by One-way Analysis of variance (ANOVA).COVID-19 Active cases are patients who had ongoing COVID-19; COVID-19 Recovered cases are patients who were previous diagnosed with COVID-19 but the subsequent test was negative by RT-PCR testing; COVID-19 Unexposed cases are subjects who have not been exposed to COVID-19, thus they have never been diagnosed with COVID-19.

### Haematological parameters among study groups

The white blood cell count (5.5±0.4 x10^9^ /L vs 4.5±0.6 x10^9^ /L vs 3.8±0.5, *p* < 0.0001) and percentage basophil (1.8±0.1% vs 0.8±0.3% vs 0.7±0.2%, *p =* 0.0040) were significantly higher among COVID-19 active cases, followed by the recovered cases and the unexposed controls. However, there was not significant difference in Eosinophil (%), Monocyte (%), Lymphocyte (%) and Neutrophil (%) between the three study groups (*p* > 0.05). With respect to red cell and platelet parameters, the COVID-19 active infection group had significantly lower levels of haematocrit (24.1±3.7% vs 38.3± 3.0% vs 38.5±2.2%, *p* < 0.0001), haemoglobin concentration (9.4±0.1g/dl vs 12.5± 5.0g/dl vs 12.7±0.8, *p* < 0.0001) and MPV (9.8±0.2fL vs 11.1±0.5fL vs 11.6±0.3fL, *p* < 0.0001) compared to recovered and unexposed controls respectively. In multiple comparison, the percentage basophil, HB, HCT and MPV were similar between the recovered patients and the unexposed healthy controls (*p* > 0.05).

Moreover, the unexposed controls had significantly higher levels of MCH and MCHC compared to the recovered and active infection groups respectively (*p*< 0.0001). There was no significant difference in red blood cell count, platelets count and MPV between the three study groups (*p* > 0.05). Table 2 displays the haematological parameters among the three study groups.

**Table 2:** Haematological parameters among the study participants.

	COVID-19 STATUS			
Parameter	Active cases	Unexposed cases	Recovered cases	*p*-value
White blood cell (x10^9^/L)	5.5±0.4	3.8±0.5	4.5±0.6	**0.0290** ^**a**^ ^**b**^ ^**c**^
Basophil (%)	1.8±0.1	0.7±0.2	0.8±0.3	**0.0040** ^**a**^ ^**c**^
Eosinophil (%)	3.6±0.4	2.5±0.5	3.2±0.8	0.3580
Monocyte (%)	7.4±0.9	11.5±2.2	12.2±2.2	0.0590
Lymphocyte (%)	45.8±2.2	49.3±3.4	40.5±2.2	0.1830
Neutrophil (%)	46.1±2.5	44.8±4.1	45.3±3.3	0.2810
Haematocrit (%)	24.1±3.7	38.5±2.2	38.3± 3.0	**<0.0001** ^**a**^ ^**c**^
Haemoglobin (g/dL)	9.4±0.1	12.7±0.8	12.5± 5.0	**<0.0001** ^**a**^ ^**c**^
Red blood cell (x10^12^/L)	4.1±0.2	4.6±0.3	4.4±0.4	0.2800
MCHC (g/L)	32.1±0.2	33.7±0.6	32.8±0.5	**<0.0001** ^**a**^ ^**b**^
MCH (pg)	28.6±0.5	31.0±0.6	28.9±0.8	**<0.0001** ^**a**^ ^**b**^
MCV (fL)	83.9±2.6	81.4±2.8	88.2±3.4	0.4410
Platelet (x10^9^/L)	227.8±84.0	276.9±55.8	280.6±49.4	0.6940
MPV (fL)	9.8±0.2	11.6±0.3	11.1±0.5	**<0.0001** ^**a**^ ^**c**^

*P-values* obtained by One-way Analysis of Variance (ANOVA) was used to compare the mean differences of haematological parameters within groups.

^a^ significant difference between active and unexposed, ^**b**^ significant difference between recovered and unexposed, ^c^ significant difference between active and recovered. MCV: Mean Corpuscular Volume, MCH: Mean Corpuscular Haemoglobin, MCHC: Mean Corpuscular Haemoglobin Concentration, MPV: Mean Platelet Volume.

### Plasma cytokine levels among the study participants

Serum IL-10 was significantly higher among COVID-19 active patients compared to unexposed controls and the recovered patients respectively (181±6.14 pg/mL vs 158.80±11.70 pg/mL vs 155.00±14.32 pg/mL, *p* = 0.038). However, IFN-γ was significantly lower among COVID-19 active patients compared to unexposed controls and the recovered patients respectively (110.10±9.52 pg/mL vs 142.80±5.46 pg/mL vs 140.80±6.39 pg/mL, *p =* 0.021). The serum levels of IL-10 and IFN-γ was similar between the unexposed controls and the recovered participants (*p* > 0.05).

**Fig 1:**
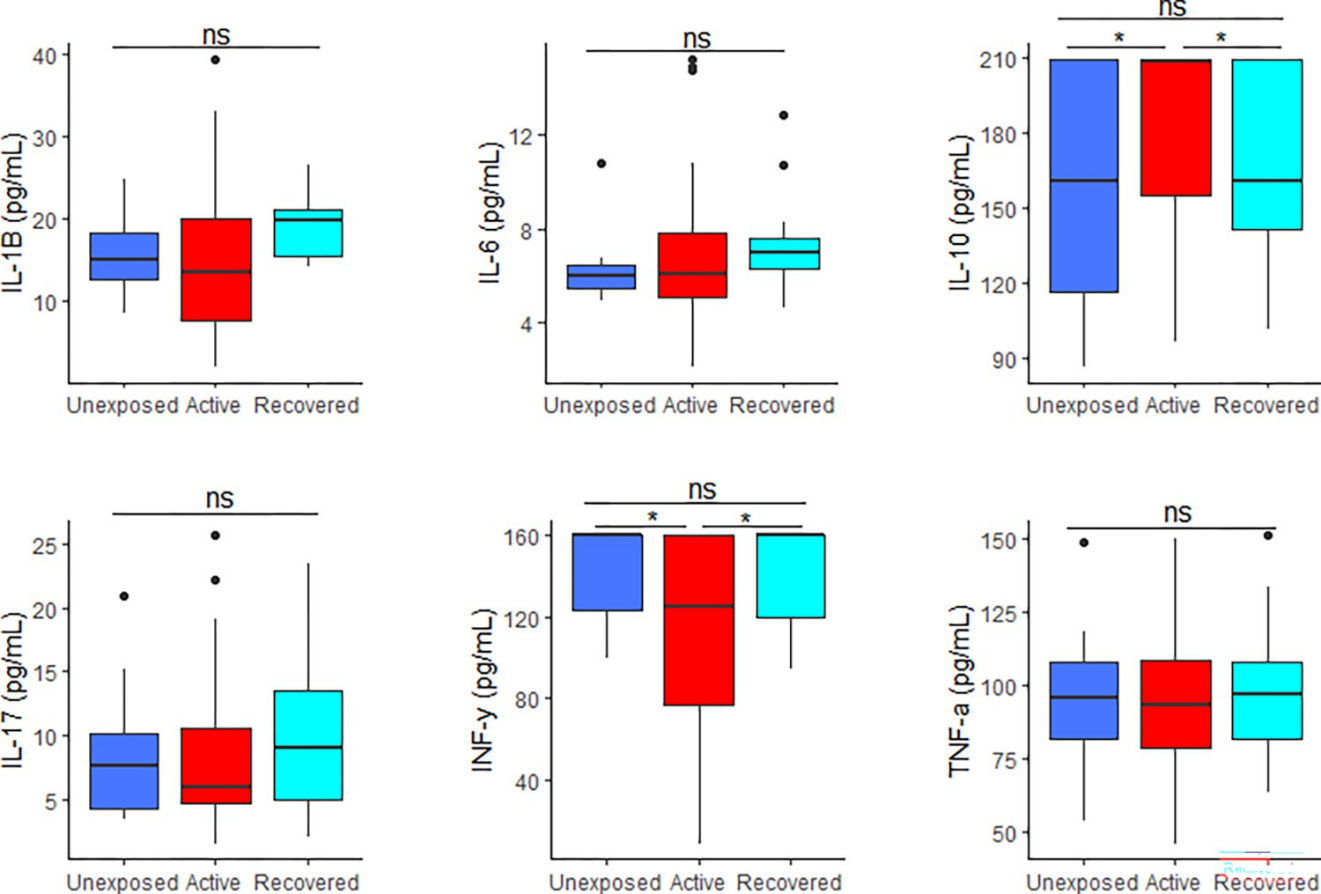
Comparison of plasma cytokine levels among the study participants.

Moreover, there was no significant difference in IL-1β (*p =* 0.937), IL-6 (*p =* 0.582), IL-17 (*p =* 0.993) and TNF-α (*p =* 0.542) between the three study groups. Fig 1 displays the cytokine levels among the three groups.

### Relationship between plasma cytokines and circulating leucocytic cell types in COVID-19

The plasma IL-1β levels in COVID-19 active cases were significantly associated with circulating neutrophils concentration (r = 0.42, p<0.05) whiles IL-1β levels in COVID-19 unexposed cases was positively associated with Lymphocytes (r = 0.57, p<0.05). The plasma IL-6 level in COVID-19 was associated with total WBC (r = 0.39, p<0.05); IL-10 levels were positive associated with WBC (r = 0.39, p<0.05) and negatively associated with Basophils (r = -0.51, p<0.01) in COVID-19 active cases; IL-17 levels in COVID-19 active cases was positively associated with Neutrophil (r = 0.39, P<0.05). Interestingly, the plasma levels of IFN-γ and TNF-ɑ among COVID-19 active cases did not show any association with circulating leucocytes. There was no association between all the plasma cytokines and circulating leucocytes among COVID-19 recovered cases. However, IL-6 was associated with Lymphocytes (r = 0.59, p<0.05); IL-10 was associated with Eosinophils (r = 0.60, p<0.05); and TNF-ɑ was associated with Basophils (r = 0.56, p<0.05), all in COVID-19 unexposed cases (Table 3).

**Table 3:** Association between cytokine levels and leucocyte parameters among the COVID-19 active cases, recovered patients and unexposed healthy controls.

	IL-1β			IL-6			IL-10			IL-17			IFN-γ			TNF-α		
Parameter	AaAct	Rec	Une	Act	Rec	Une	Act	Rec	Une	Act	Rec	Une	Act	Rec	Une	Act	Rec	Une
White blood cell	0.35	0.16	-0.02	0.39*	0.18	-0.10	**0.39** *	0.14	0.02	0.30	0.15	-0.04	0.05	0.44	-0.24	0.1	-0.43	-0.19
Basophil	-0.21	0.25	0.03	-0.21	0.18	-0.02	**-0.51** **	-0.00	0.19	-0.20	0.20	0.03	0.1	-0.61	0.00	-0.02	0.3	**0.56** *
Eosinophil	-0.16	-0.19	0.08	-0.15	-0.17	0.21	0.08	-0.12	0.60*	-0.13	-0.26	0.13	-0.25	0.2	0.07	0.05	0.16	0.42
Monocyte	-0.22	-0.2	-0.05	-0.29	-0.23	-0.06	-0.38	0.11	0.10	-0.21	-0.2	-0.07	0. 06	0.42	-0.22	-0.1	-0.1	-0.01
Lymphocyte	-0.33	0.09	**0.57** *	-0.27	0.12	0.59*	0.04	-0.41	0.22	-0.3	0.01	**0.60** *	-0.09	-0.4	-0.12	0.05	0.35	0.26
Neutrophil	**0.42** *	0.07	-0.46	0.35	0.06	-0.49	0.11	0.18	-0.32	0.39*	0.12	-0.49	0.18	-0.04	0.2	0.01	-0.24	-0.3

Pearson correlation coefficients between cytokines and the five main types of leucocytes cells among Covid-19 active cases, recovered cases, Negative (unexposed) cases

(*p ≤ 0.05

** p ≤ 0.01), Act: Covid-19 active cases; Rec, Covid-19 recovered cases; Une, Covid-19 unexposed cases.

## Discussion

COVID-19 has been reported to induced total leucocyte and neutrophil counts with severe lymphopenia as an early sign of disease complication [8, 26]. The lymphopenia, neutrophil/lymphocyte ratio (NLR) are considered as potential diagnostic criteria for severe COVID-19 cases [34]. This indicates that peripheral blood counts may play an essential role in the progressing of the COVID-19 infectious disease [35, 36]. The striking morphologic changes and intensity of peripheral WBCs, neutrophilia and lymphopenia between mild and severe COVID-19 diseases have been demonstrated [24]. However, the immunohaematological profile following recovery from COVID-19 has not gained much interest. We assessed the evaluated the haematological profile and the plasma cytokine levels among COVID-19 active patients, recovered cases and unexposed healthy controls.

The result showed that total circulating WBC and basophils were significantly higher among COVID-19 active cases and the recovered patients compared to the unexposed healthy controls. The macrophage proliferation and increased macrophages are the predominant inflammatory cells that have been observed in alveolar injuries in the severe COVID-19 cases [37, 38]. Antibodies produced against COVID-19 spike glycoprotein has been reported to induced infiltration and accumulation of proinflammatory monocytes and macrophages in the lungs where virus-mediated lung injury causes [15, 39]. The circulating basophils number has been reported to correlate with IgG response in COVID-19, suggesting the potentiation and enhancement effects of basophil to humoral response to COVID-19 [40, 41]. Increased basophils circulation has been associated with a lower risk of developing severe COVID-19 [40, 42]. Basophil regulates innate immune response to COVID-19 and repairs damaged tissues caused by inflammation [43, 44]. All the COVID-19 active cases enrolled on the study were experiencing a mild form of infection. This explains the high number of circulating basophils among the active infection cases compare to the recovered cases and the unexposed COVID-19 cases [37, 41]. Moreover, the levels of circulating basophils was similar between the recovered patients and the unexposed healthy controls suggesting that patients have innate immune recovery with apparently normal white production of the WBC subsets.

The COVID-19 active infection group had significantly lower levels of haematocrit, HB, and MPV compared to the recovered patients and unexposed controls. This results is similar to the finding of Djakpo et al. [45]. Several previous studies have reported low haemoglobin and HCT and difference in the red cell indices (MCV, MCH and MCHC) between mild and severe COVID-19 cases, suggesting changes in these observed parameters are critical monitoring and administration of appropriate management when the need arises [24, 46–48]. Although there are significant difference in the red cell indices among the study groups, all the values were within the normal standard reference ranges suggesting that the low HB and HCT observed in our COVID-19 patients may be due to haemolysis associated with the complex disseminated intravasxular couagulation (DIC) in the pathophysiology of COVID-19 where there is normocytic normochromic anaemia [49, 50]. Again, it has been established that COVID-19 non-structural spike proteins exert inhibitory effects on the haemoglobin by binding to heme and cause an imbalance in iron metabolism [51–53]. Hence, COVID-19 effects on the erythrocyte parameters have not been observed in the pathological cases of the COVID-19. However, the COVID-19 patients had mild form of the disease explaining why we found normal red cell indices.

The antiinflammatory cytokine, IL-10 has been identified with protective roles in COVID-19. In the current study, IL-10 was significantly higher among patients with active COVID-19 conpared to the recovered patients and the unexposed controls. The increased IL-10 concentration in the plasma sample of active COVID-19 cases indicates providing some form of protection to the infected individuals [53, 54]. IL-10 is a nonspecific cell-type cytokine, widely expressed by several immune cells with many layers of regulating its production [55]. IL-10 is mainly produced by macrophages, dendritic cells and CD4+ T cells [56, 57]. IL-10 production is triggered by pathogen activation of DCs and macrophages that are involved in the recognition of pathogen-derived products by pattern recognition receptors. Aside these cells, CD8+ T cells also produce IL-10 following TCR activation [58]. Neutrophils have been reported to express IL-10 [59]. Agonists to Toll-like receptor 2 (TLR2) are specialized in inducing IL-10 expression by APCs [59]. Example; TLR2 influence IL-10 production by macrophages following pneumoccocal cell wall stimulation [60]. Again, higher amounts of IL-10 are also produced by macrophages and myeloid DCs following stimulation with TLR4 and TLR9 ligands [61]. The higher levels of IL-10 among patients with active COVID-19 suggests activated cellular non-specific cellular production by IL-10 producing cells or high ligand action of TLRs. The apprantly normal levels of IL-10 in recovered patients and healthly controls could be due to less activation by IL-10 producing cells as well as TLR ligans. IL-10 inhibit TNF-ɑ and neutrophil activation in an acute lung injury [2, 62]. Although dramatic elevation of IL-10 and IL-6 with inflammatory markers such as C-reactive protein have been reported in severe or critical COVID-19 cases [63–65]. All the COVID-19 active cases had the mild disease with no evidence of increased IL-6 levels compared among the recovered and health groups. This suggests that the increased levels of IL-10 may be suppressing inflammation through a negative feedback mechanism. Moreover, IL-10 was similar among the recovered patients and the unexposed healthy controls. This suggests that recovered patients have achieved imune recovery with less production of antiinflammatory cytokines.

Decrease IFN-γ expression in CD4+ T cells has been associated with severe COVID-19 cases in severe reports [66, 67]. However, an increase in IFN-γ levels in the peripheral blood had also been reported in severe cases compared to mild cases [34]. In our study, plasma IFN-γ levels were low among patients with active infection compared to the recovered patients and the unexposed control groups. The low IFN-γ was detected in mild active cases suggesting that the increased IL-10 may be suppressing pro-inflammatory cytokines through a negative feedback mechanism [68]. This probably prevents the overactivation of inflammatory cells such as macrophages as observed in cytokine storm syndrome [9].

Basophil-mediated IL10 induction has been reported to diminish without IL-6 or IL-4, however, culturing antigen-presenting cells (APCs) or CD8 with either IL-6 or IL-4 could not sufficiently secrete IL-10 production [69, 70]. But in the presence of IL-6 and IL-4 without basophils significantly induced IL-10 production [70, 71]. This suggested that basophils only present antigens to CD8+ T cells to induce IL-10 production [70]. This explains the negative correlations observed between IL-10 and basophils in COVID-19 active cases. Also, the study observed a positive association between neutrophils and each of plasma IL-1β and IL-17 in the active COVID-19 cases. Both IL1-β and IL-17 stimulation from the Th1/Th17 response increases the vascular permeability which results in the recruitment and infiltration of neutrophils in the affected sites and forms NETs and NETosis to clear the virus infections.

Finally, the observed increased levels of IL-10, neutrophils and basophils with a conconmittant decrease in IFN-γ may had confers some level of protections to the Ghanaians by preventing the development of severe or critical disease cases in mild COVID-19 resulting higher prevelance of mild active cases of COVID-19 among Ghanaian population.

Our study had few limitations. First, all the COVID-19 patients were diagnosed with the mild form of the disease and could not explore into how the variable differe in severe disease compared prior to recovery. Again, the study was conducted with a relatively small sample size which may have some impact on the statistical comparisons. Confirming our findings in larger prospective cohort studies may contribute to better understanding of COVID-19 following reovery.

## Conclusison

COVID-19 active infection is associated with increased IL-10 and WBC with a conconmittant decrease in IFN-γ and haemoglobin concentation. However, recovery from the disease is associated with immune recovery with appreantly normal haematological profile.

## Supporting information

S1 Data (XLSX)
